# Schwertmannite Synthesis through Ferrous Ion Chemical Oxidation under Different H_2_O_2_ Supply Rates and Its Removal Efficiency for Arsenic from Contaminated Groundwater

**DOI:** 10.1371/journal.pone.0138891

**Published:** 2015-09-23

**Authors:** Fenwu Liu, Jun Zhou, Shasha Zhang, Lanlan Liu, Lixiang Zhou, Wenhua Fan

**Affiliations:** 1 Environmental Engineering Laboratory, College of Resource and Environment, Shanxi Agricultural University, Taigu, China; 2 College of Biotechnology and Pharmaceutical Engineering, Nanjing Tech University, Nanjing, China; 3 Department of Environmental Engineering, College of Resources and Environmental Sciences, Nanjing Agricultural University, Nanjing, China; Institute for Materials Science, GERMANY

## Abstract

Schwertmannite-mediated removal of arsenic from contaminated water has attracted increasing attention. However, schwertmannite chemical synthesis behavior under different H_2_O_2_ supply rates for ferrous ions oxidation is unclear. This study investigated pH, ferrous ions oxidation efficiency, and total iron precipitation efficiency during schwertmannite synthesis by adding H_2_O_2_ into FeSO_4_·7H_2_O solution at different supply rates. Specific surface area and arsenic (III) removal capacity of schwertmannite have also been studied. Results showed that pH decreased from ~3.48 to ~1.96, ~2.06, ~2.12, ~2.14, or ~2.17 after 60 h reaction when the ferrous ions solution received the following corresponding amounts of H_2_O_2_: 1.80 mL at 2 h (treatment 1); 0.90 mL at 2 h and 14 h (treatment 2); 0.60 mL at 2, 14, and 26 h (treatment 3); 0.45 mL at 2, 14, 26, and 38 h (treatment 4), or 0.36 mL at 2, 14, 26, 38, and 50 h (treatment 5). Slow H_2_O_2_ supply significantly inhibited the total iron precipitation efficiency but improved the specific surface area or arsenic (III) removal capacity of schwertmannite. For the initial 50.0 μg/L arsenic (III)-contaminated water under pH ~7.0 and using 0.25 g/L schwertmannite as an adsorbent, the total iron precipitation efficiency, specific surface area of the harvested schwertmannite, and schwertmannite arsenic(III) removal efficiency were 29.3%, 2.06 m^2^/g, and 81.1%, respectively, in treatment 1. However, the above parameters correspondingly changed to 17.3%, 16.30 m^2^/g, and 96.5%, respectively, in treatment 5.

## Introduction

Arsenic, a metalloid of Group VA in the periodic table, has four valence states: +5, +3, 0, and -3 [1]. However, arsenic (V) and arsenic (III) are the predominant forms in the environment [2]. In general, arsenic can be found in inorganic or organic forms. However, the abundance of organic arsenic is significant only in waters with marked impact of industrial pollution [3]. This element is a major poisonous inorganic pollutant in groundwater, especially in rural endemic arsenic poisoning areas [4]. In addition, arsenic is significant present as arsenite in groundwater under reducing conditions at pH below 9.0 [1, 2].

Millions of people in many countries, such as China, India, Nepal, and Bangladesh, are suffering from arsenicosis because of their long-term exposure to drinking groundwater contaminated with high levels of arsenic [5, 6, 7, 8]. Arsenic concentrations were ranged from 0 to 100 μg/L in more than 90% of 31,667 groundwater samples in Nawalparasi and Rupandehi located in Nepal [6]. In China, over 2.3 million people in Shanxi Province, Inner Mongolia, Xinjiang, Ningxia, Jilin Province, and Anhui Province are affected by arsenicosis, and more than 0.5 million people are drinking arsenic-contaminated water with arsenic concentrations exceeding 50 μg/L [9]. Arsenic concentration in groundwater has the range from 0.1 to 116 μg/L in the Taiyuan basin, lying in the center of the Shanxi province in China [10]. Therefore, the removal of arsenic from contaminated water and the provision of safe and economically affordable drinking water are challenging.

Adsorption is a simple, efficient, and low-cost method to purify water [11]. Activated carbon [12], fly ash [13], bio-wastes [14], and iron- or aluminum-based materials (i.e., ferrihydrite, ferric hydroxide, iron-aluminum hydroxide, and activated alumina) [15] show remarkable potential toward arsenic removal. However, iron-based substances have emerged as treatment adsorbents for arsenic removal from contaminated water [1].

Schwertmannite is an iron-based amorphous iron-oxyhydroxysulfate mineral commonly reported as a brownish yellow precipitate in iron- and sulfate-rich acidic environment [16, 17]. The main reaction equation is as follows:

$$4{\text{Fe}}^{2+}+{\text{O}}_{{}^{2}}+4{\text{H}}^{+}\overset{}{\to }4{\text{Fe}}^{3+}+2{\text{H}}_{2}\text{O}$$ (1)

$$8{\text{Fe}}^{3+}+{\text{xSO}}_{4}{}^{2\text{-}}+(16\;\text{-}\;2\text{x}){\text{H}}_{2}\text{O}\to {\text{Fe}}_{8}{\text{O}}_{8}{\left(\text{OH}\right)}_{8}{}_{\text{-}2\text{x}}{\left({\text{SO}}_{4}\right)}_{\text{x}}(\text{schwertmannite})+(24\;\text{-}\;2\text{x}){\text{H}}^{+}$$ (2)

Schwertmannite is an efficient scavenger of arsenic from arsenic-contaminated waters [18, 19], and arsenic retention is mainly controlled by ligand exchange with adsorbed SO_4_^2-^ or formation of amorphous As-Fe(III)-SO_4_^2-^ precipitates on the schwertmannite surface [20, 21, 22]. Therefore, the specific surface area of schwertmannite is an important indicator of its arsenic adsorption capacity [23, 24]. In general, schwertmannite with a large specific surface area has a great arsenic adsorption capacity [21]. For example, the maximum arsenic (III) adsorption capacity of schwertmannite with a specific surface area of 325.5 m^2^/g is 45.50 mg/g at pH 3.0 [23], whereas that of schwertmannite with a specific surface area of 210.0 m^2^/g is only 20.1 mg/g [21]. However, limited studies have been performed on arsenic removal using schwertmannite under neutral pH groundwater conditions.

Schwertmannite can be synthesized through several techniques, such as addition of ferric chloride / nitrate into sodium / potassium sulfate solutions at 60°C [25], ferric sulfate hydrolysis at 85°C [26], and chemical or biogenic oxidation of FeSO_4_ solutions by H_2_O_2_ or *Acidithiobacillus ferrooxidans* (*A*. *ferrooxidans*) prior to ferric ions hydrolysis [27, 28]. Among these technologies, the oxidation of FeSO_4_ by H_2_O_2_ or *A*. *ferrooxidans* has received the most attention because of the low power consumption of this method during solution heating. Compared with *A*. *ferrooxidans*, H_2_O_2_ is preferred for oxidizing ferrous ions in FeSO_4_ solution because the synthesis conditions are more likely to be controlled without considering the factors of microorganisms growth. Regenspurg et al. [27] reported that the specific surface area of rapidly crystallized schwertmannite is 4~14 m^2^/g when schwertmannite synthesized through the H_2_O_2_ oxidation of FeSO_4_ solutions. Similarly, Paikaray et al. [21] or Li et al. [29] reported that the specific surface area of schwertmannite synthesized using the same method is 5.3 or 3.2 m^2^/g, respectively. However, the factor that controls the specific surface area of schwertmannite during the H_2_O_2_ oxidization of ferrous ions remains unknown. The chemical oxidation of ferrous ions under acidic conditions produces ferric ions, some of which are hydrolyzed and precipitated as schwertmannite. Therefore, ferrous ions oxidation efficiency, which is controlled by H_2_O_2_ supply rate, possibly influences the total iron precipitation efficiency or the specific surface area of synthesized schwertmannite, and thus schwertmannite arsenic adsorption. However, insufficient information is available about this topic.

This study investigated the influence of H_2_O_2_ supply rate on the total iron precipitation efficiency in system or specific surface area of schwertmannite synthesized through the H_2_O_2_ oxidization of FeSO_4_ solutions. It also determined the effects of schwertmannite harvested from different H_2_O_2_ supply rate systems on arsenic adsorption capacity under neutral pH. The results of this study may serve as a basis in understanding the synthesis behavior and the arsenic-removal capacity of schwertmannite under the reducing condition of groundwater aquifer.

## Materials and Methods

The reagents and chemicals used in the experiments were all of analytical grade, and all solutions were prepared fresh with deionized water when required. All laboratory glass and plastic wares were conditioned by 10% HNO_3_ overnight and rinsed several times with deionized water before use.

### Schwertmannite chemical synthesis

Schwertmannite was obtained using the H_2_O_2._ oxidizing ferrous ions solution. The pre-experiments showed that the ferrous ions were completely oxidized when 1.80 mL of 30% (V/V) H_2_O_2_ was added into 150 mL of 160 mmol/L FeSO_4_·7H_2_O solution. In this study, H_2_O_2_ were added into 150 mL of 160 mmol/L FeSO_4_·7H_2_O solution as following method: 1.80 mL at 2 h (treatment 1); 0.90 mL at 2 h and 14 h (treatment 2); 0.60 mL at 2, 14, and 26 h (treatment 3); 0.45 mL at 2, 14, 26, and 38 h (treatment 4); or 0.36 mL at 2, 14, 26, 38, and 50 h (treatment 5). During schwertmannite synthesis under the different treatments, the pH, ferrous ions oxidation efficiency, and total iron precipitation efficiency were monitored at 2, 12, 14, 24, 26, 36, 38, 48, 50, and 60 h. After 60 h, the precipitates that formed after the different treatments were collected through filtering with Whatman No. 4 filter paper and oven-dried at 50°C to constant weight. The dried solid precipitates were evaluated for their mineral phases, morphology, specific surface area, and mineral chemical composition. All treatments were designed with four replicates.

### Arsenic (III) adsorption removal experiments

The 1000 mg/L arsenic (III) stock solution was prepared by dissolving 0.6595 g dehydrated arsenic oxide (As_2_O_3_) power in 500 mL of 0.04 mol/L NaOH solution since As_2_O_3_ has enhanced solubility in NaOH solution. Arsenic (III) adsorption removal experiments were conducted by adding 10 mg of different chemically synthesized schwertmannite harvested from the above experiments to 100 mL capped conical flasks each containing 40 mL of solution comprising of 25, 50, or 100 μg/L initial As(III) obtained by dilution of stock solution. The suspension pH was adjusted to ~7.0 by adding 0.1 mol/L HCl or NaOH dropwise. All flasks were shaken for 24 h in a reciprocating shaker at 180 r/min and 30°C. After 24 h, the solution was filtered through a 0.22 μm membrane and examined for arsenic concentration. All treatments were conducted with four replicates.

### Analytical procedures

Solution pH was measured using a pHS-3C model digital pH-meter. Ferrous ions or total iron concentration was determined by using the 1, 10-phenanthroline method [2]. The ferrous ions oxidation efficiency or the total iron precipitation efficiency was calculated using the following formula: ferrous ions oxidation efficiency (%) = [*(C*_*0*_ *-C*_*t*_ *)/C*_*0*_]×100%, where *C*_*o*_ is the initial ferrous ions concentration, and *C*_*t*_ is the ferrous ions concentration at different times after treatments. The total iron precipitation efficiency was calculated using the following formula: total iron precipitation efficiency (%) = [(*C*_*0*_^*’*^ *-C*_*t*_^*’*^ *)/C*_*o*_^*’*^)]×100%, where *C*_*o*_^*’*^ is the initial total iron concentration, and *C*_*t*_^*’*^ is total iron concentration at different times after treatments [30]. The mineral phase or morphology of precipitates was determined through power X-ray diffraction (XRD, MiniFlex II, Japan) using CuKα radiation (30KV, 15mA) or field-emission scanning electron microscopy (SEM, JSM-7001F) [31]. The specific surface area of the precipitate was determined using the Brunauer-Emmett-Teller (BET) method by adsorption of N_2_ gas at liquid N_2_ temperature using automatic specific surface and porosity analyzer (TriStar II 3020) [32]. Total arsenic in solution was determined through atomic fluorescence spectrophotometry (HG-AFS, 230E) [8] with a detection limit of 0.007 μg/L. To identify the chemical composition of the synthesized solids, 0.1 g of the precipitates were dissolved in 100 mL of 0.1 M HCl, and the Fe and S contents were determined through ICP-AES [33].

### Statistical analysis

Experimental data has been analyzed by SAS 9.2 software. All data shown in Figures are the mean values of four replicates with standard deviations to show their reproducibility and reliability.

## Results and Discussion

### Changes in pH during schwertmannite chemical synthesis

**Fig 1:**
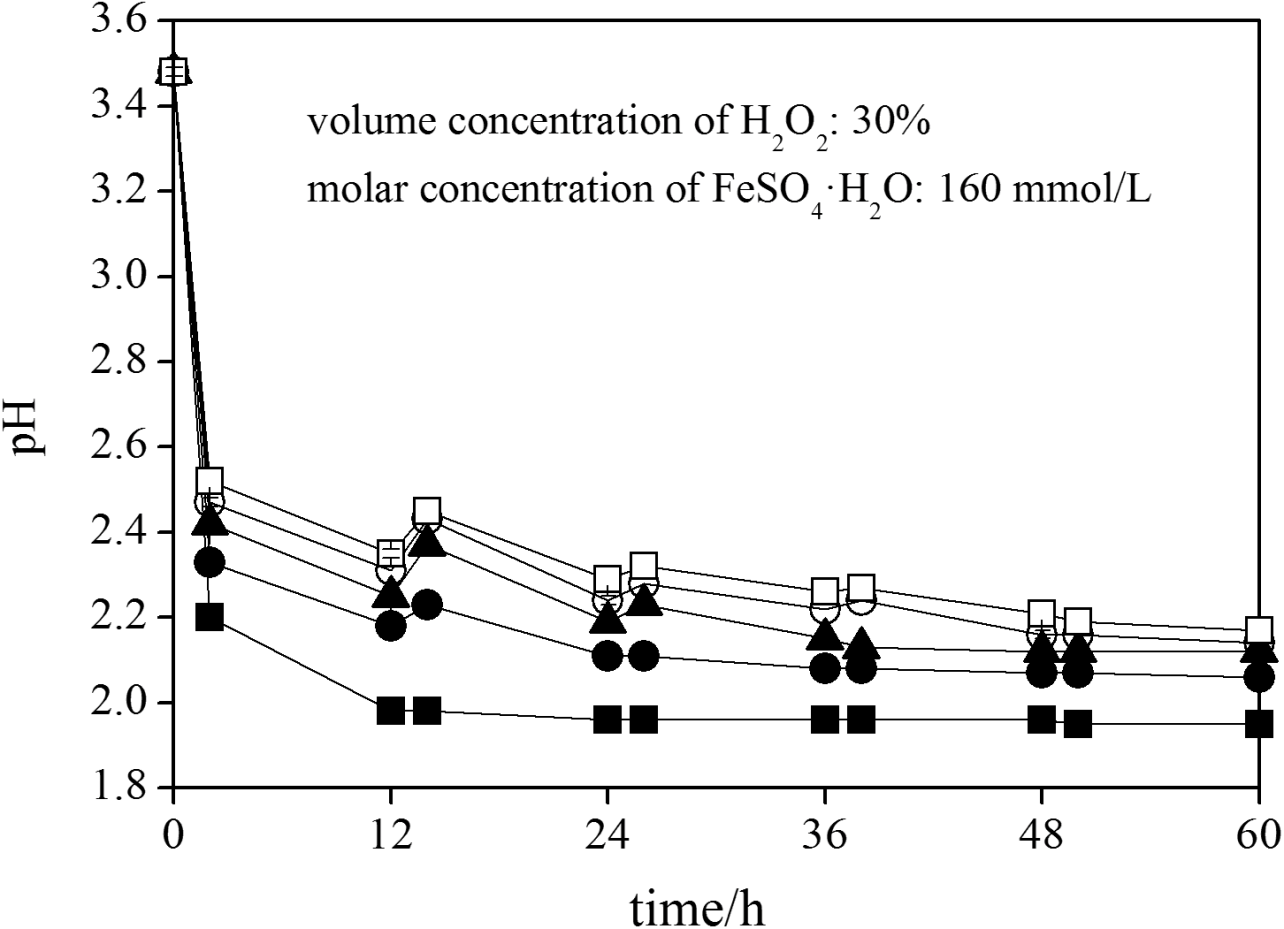
Change in pH during ferrous ions chemical oxidation with H_2_O_2_ adding FeSO_4_·7H_2_O solution at different rates. 150 mL of FeSO_4_·7H_2_O solution with addition of ■: 1.80 mL of H_2_O_2_ at 2 h (treatment 1); ●: 0.90 mL of H_2_O_2_ at 2 and 14 h (treatment 2); ▲: 0.60 mL of H_2_O_2_ at 2, 14, and 26 h (treatment 3); ○: 0.45 mL of H_2_O_2_ at 2, 14, 26, and 38 h (treatment 4); □: 0.36 mL of H_2_O_2_ at 2, 14, 26, 38, and 50 h (treatment 5).

Ferrous ions can be oxidized to ferric ions in the presence of H_2_O_2,_ during which the pH of the solution may increase [34]. However, the pH in the solution can also be decreased because of the release of H^+^ resulting from schwertmannite formation [35]. In this study, the changes in pH during schwertmannite chemical synthesis with different H_2_O_2_ supply rates for ferrous ions oxidation are illustrated in Fig 1.

The initial pH of the FeSO_4_·7H_2_O solution was ~3.48. The pH sharply decreased to ~2.20 after 2 h, slightly decreased to ~1.98 after 12 h, and then remained stable at ~1.96 until the completion of the experiment in treatment 1. Similar to the treatment 1, the pH decreased from initial ~3.48 to ~2.33, ~2.42, ~2.47, and ~2.52 at 2 h because of H_2_O_2_ addition at this point in treatment 2, treatment 3, treatment 4, and treatment 5. However, expect for H_2_O_2_ added point at 2 h, the pH has an slight increase trend in treatment 2–5 at H_2_O_2_ added points because of H^+^ ions consumed due to ferrous ions oxidation more than H^+^ produced due to schwertmannite formation at these time points. For instance, the system pH at 14 h (pH ~2.23, H_2_O_2_ adding point) higher than it at 12 h (pH ~2.18) in treatment 2. In treatment 2, the pH remarkably decreased from ~3.48 to ~2.33 at 2h, gradually decreased to ~2.18 or ~2.08 at 12 or 36 h, and then remained relatively stable at ~2.06 until the termination of the schwertmannite synthesis trial. The solution pH initially sharply declined and then slightly decreased during the schwertmannite synthesis trials in all treatments. The solutions pH continuously decreased from initial ~3.48 to ~2.12, ~2.14, and ~2.17 within 60 h of in treatment 3, treatment 4, and treatment 5, respectively.

It is found that the fast H_2_O_2_ supply for ferrous ions oxidation, or fast ferric ions supply for schwertmannite synthesis, can resulted in low solution pH after the experiment. This phenomenon agrees with the results obtained by Liu et al. [36] and Zhu et al. [35] during the ferrous ions bio-oxidation by *A*. *ferrooxidans* for iron oxyhydroxysulfate mineral formation. They found that the significant decline in solution pH efficiency depends on the rate of ferric ions supply, which is controlled by the incubation density or oxidation ability of *A*. *ferrooxidans* [35, 36] in systems. Moreover, the increase of pH was not observed when ferrous ions were rapidly oxidized, such as in treatment 1. This finding is consistent with the results obtained by Wang and Zhou [37], who found that the pH gradually decreases from 2.50 to 1.80 without increasing trend during *A*. *ferrooxidans* incubation for iron oxyhydroxysulfate mineral bio-synthesis under rapid ferrous ions bio-oxidation. The increase in pH was quickly counteracted by the subsequent ferric ions hydrolysis with more H^+^ release when the ferrous ions were rapidly oxidized to ferric ions through chemical or biological oxidation.

### Variations in ferrous ions oxidation efficiency during schwertmannite chemical synthesis

**Fig 2:**
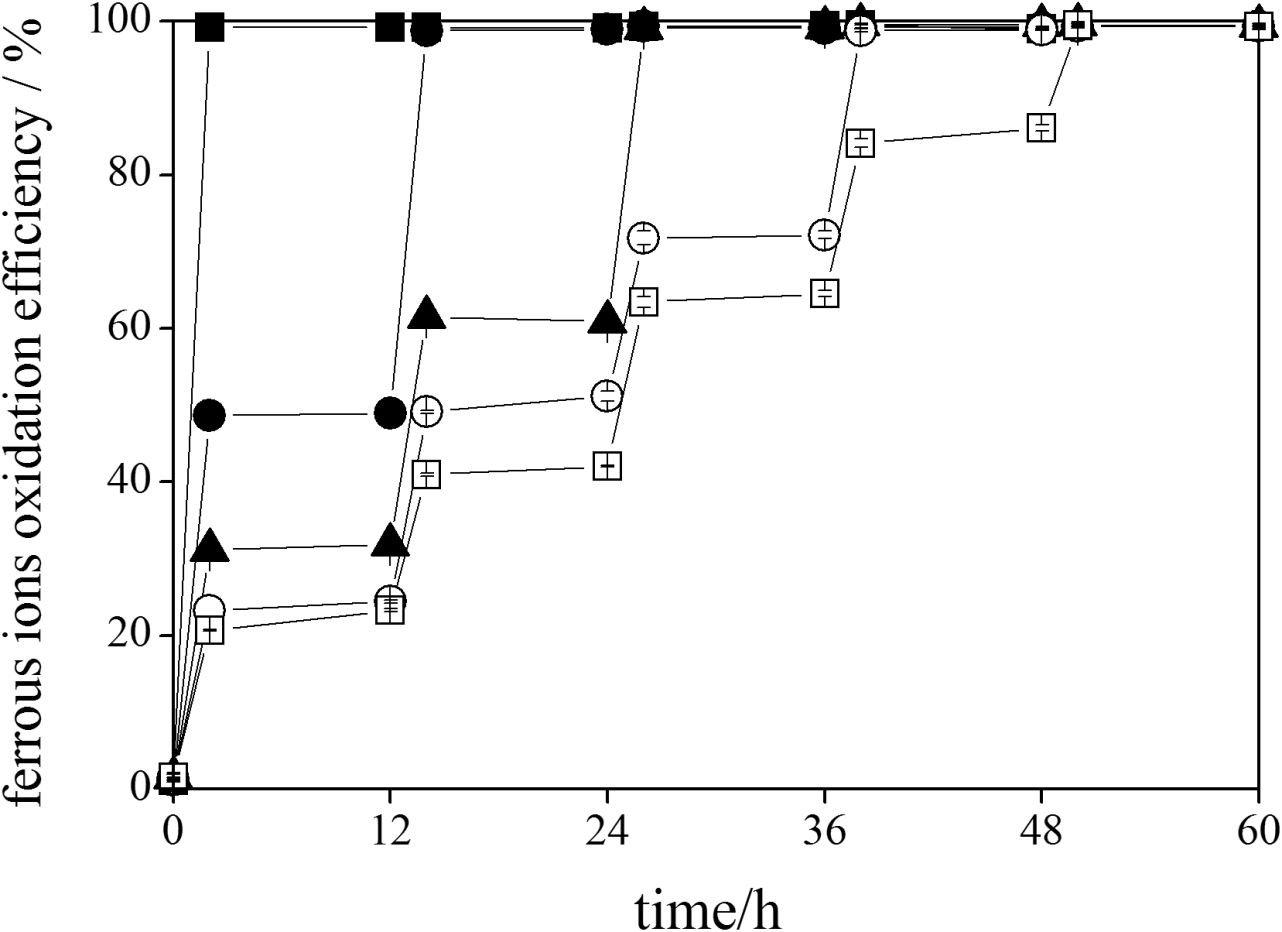
Variations in ferrous oxidation efficiency during ferrous ions chemical oxidation with H_2_O_2_ adding FeSO_4_·7H_2_O solution at different rates. 150 mL of FeSO_4_·7H_2_O solution with addition of ■: 1.80 mL of H_2_O_2_ at 2 h (treatment 1); ●: 0.90 mL of H_2_O_2_ at 2 and 14 h (treatment 2); ▲: 0.60 mL of H_2_O_2_ at 2, 14, and 26 h (treatment 3); ○: 0.45 mL of H_2_O_2_ at 2, 14, 26, and 38 h (treatment 4); □: 0.36 mL of H_2_O_2_ at 2, 14, 26, 38, and 50 h (treatment 5).

The oxidation of ferrous ions to ferric ions is the first and the rate-limiting step during the formation of schwertmannite under acidic and sulfate-rich environment [38, 39]. In the present study, variations in the efficiency of ferrous ions oxidation during schwertmannite chemical synthesis with different H_2_O_2_ supply rates for ferrous ions oxidation are given in Fig 2.

The ferrous ions were completely oxidized by H_2_O_2_ in all treatments, and ferrous ions oxidation efficiency was mainly depend on the added amount of H_2_O_2_ (Fig 2)_._ Ferrous ions oxidation efficiency immediately reached 99.2% in treatment 1. In treatment 2, when 0.90 mL of H_2_O_2_ was added into system at 2h and 14 h, the ferrous ions oxidation efficiency were 48.7% and 98.8% at 2 h and 14 h. Similarly, the ferrous ions oxidation efficiency reached 31.2%, 61.5%, and 99.1% at 2 h, 14 h and 36 h when 0.60 mL of H_2_O_2_ was added into schwertmannite synthesis system at the above three time points in treatment 3. A similar variation trend was also observed in the other treatments. The ferrous ions oxidation efficiency was completely reached at 38 h and 50 h in treatment 4 and treatment 5, respectively. The data in Fig 2 clearly showed the slight oxidation efficiency of ferrous ions during the reaction between the the H_2_O_2_ addition points. For example, the ferrous ions oxidation efficiency slowly increased from 23.3% to 24.5%, 49.1% to 51.2%, and 71.8% to 72.2% during the 2~12 h, 14~24 h, and 26~36 h reaction periods respectively during the chemical synthesis of schwertmannite in treatment 4. Ferrous ions oxidation depends upon the oxidation potential of the oxidant, which is lower for oxygen (0.401 V), and significantly higher for H_2_O_2_ (1.77 V) [40]. Therefore, the efficiency of ferrous ions oxidation by using H_2_O_2_ is much higher than by using oxygen. Moreover, Johnson and Hallberg [41] reported that the abiotic oxidation of ferrous iron is very slow at pH<4.0 even in oxygen-saturated waters. In this study, the schwertmannite synthesis in all treatments was conducted below pH 3.48. Therefore, the ferrous ions oxidized to ferric ions were mainly dependent on the oxidizing agent H_2_O_2_ rather than on oxygen. The color of schwertmannite synthesis systems changed from light green to reddish brown with ferrous ions oxidation, and some orange-colored precipitates were observed in different treatments.

### Variations in the total iron precipitation efficiency during schwertmannite chemical synthesis

**Fig 3:**
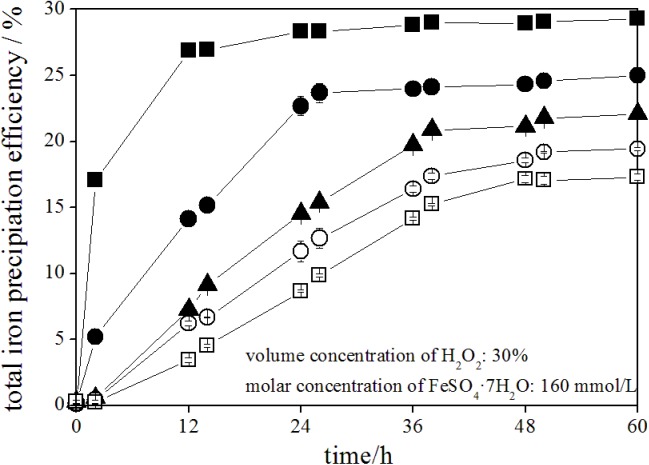
Variations in total Fe precipitation during ferrous ions chemical oxidation with H_2_O_2_ adding FeSO_4_·7H_2_O solution at different rates. 150 mL of FeSO_4_·7H_2_O solution with addition of ■: 1.80 mL of H_2_O_2_ at 2 h (treatment 1); ●: 0.90 mL of H_2_O_2_ at 2 and 14 h (treatment 2); ▲: 0.60 mL of H_2_O_2_ at 2, 14, and 26 h (treatment 3); ○: 0.45 mL of H_2_O_2_ at 2, 14, 26, and 38 h (treatment 4); □: 0.36 mL of H_2_O_2_ at 2, 14, 26, 38, and 50 h (treatment 5).

Oxidation of ferrous ions under acidic conditions produced ferric ions, some of which hydrolyzed and precipitated as iron oxyhydroxysulfate mineral [17, 30], such as schwertmannite in this study. In other words, higher efficiency of total iron precipitation corresponds to more synthetic schwertmannite. Fig 3 shows the total iron precipitation efficiency during schwertmannite chemical synthesis in all treatments.

Total iron precipitation efficiency sharply increased to 17.0%, 26.9%, and slightly to 29.3% at 2, 12, and 60 h, respectively, during schwertmannite synthesis in treatment 1. In treatment 2, when 0.90 mL of H_2_O_2_ was added into schwertmannite synthesis system at 2h and 14 h, the total iron precipitation efficiency increased to 5.2%, 15.2%, 22.7% and slightly to 25.0% at 2, 14, 24, and 60 h, respectively. Similarly, when 0.60 mL of H_2_O_2_ was added into schwertmannite synthesis system at 2 h, 14 h, and 26 h, the total iron precipitation efficiency increased to 0.4%, 6.7%, 12.7%, 16.4% and slightly to 19.4% at 2, 14, 26, 36, and 60 h, respectively. It is noted that iron precipitation behavior mainly occurred at H_2_O_2_ addition point or within 10 h after addition. When 10 hours after ferrous ions oxidation completely, the total iron precipitation efficiency showed no obvious increase with the extension of time. Similar trends have been observed in all treatments. In the experiment by Liao et al. [28], the efficiency of ferric ions precipitation rapidly increased to 37.7% at 16 h, and then slowly increased to ~40.5% until 40 h when the ferrous ions was completely oxidized by *A*. *ferrooxidans* at 16 h during schwertmannite bio-synthesis in the acidic tannery sludge bioleach solution. It is concluded that ferrous ions oxidation and total iron precipitation reactions are dynamically interrelated in ferrous ions chemical or biological systems. After the termination of schwertmannite chemical synthesis trials, the total iron precipitation efficiency reached 22.1%, 19.4%, and 17.3% in treatment 3, treatment 4 and treatment 5, respectively. Rapid H_2_O_2_ supply corresponds to fast ferrous ions oxidation efficiency and high total iron precipitation efficiency in the schwertmannite synthesis systems. This phenomenon had not been reported during schwertmannite chemical synthesis by H_2_O_2_ oxidizing ferrous ions in previous studies. However, Wang and Zhou [37] reported similar results in iron oxyhydroxysulfate mineral jarosite bio-synthesis systems. They concluded that the total iron precipitation efficiency significantly improves with ferrous ions bio-oxidation efficiency.

### Phase and morphology of solid precipitates harvested from schwertmannite chemical synthesis systems

**Fig 4:**
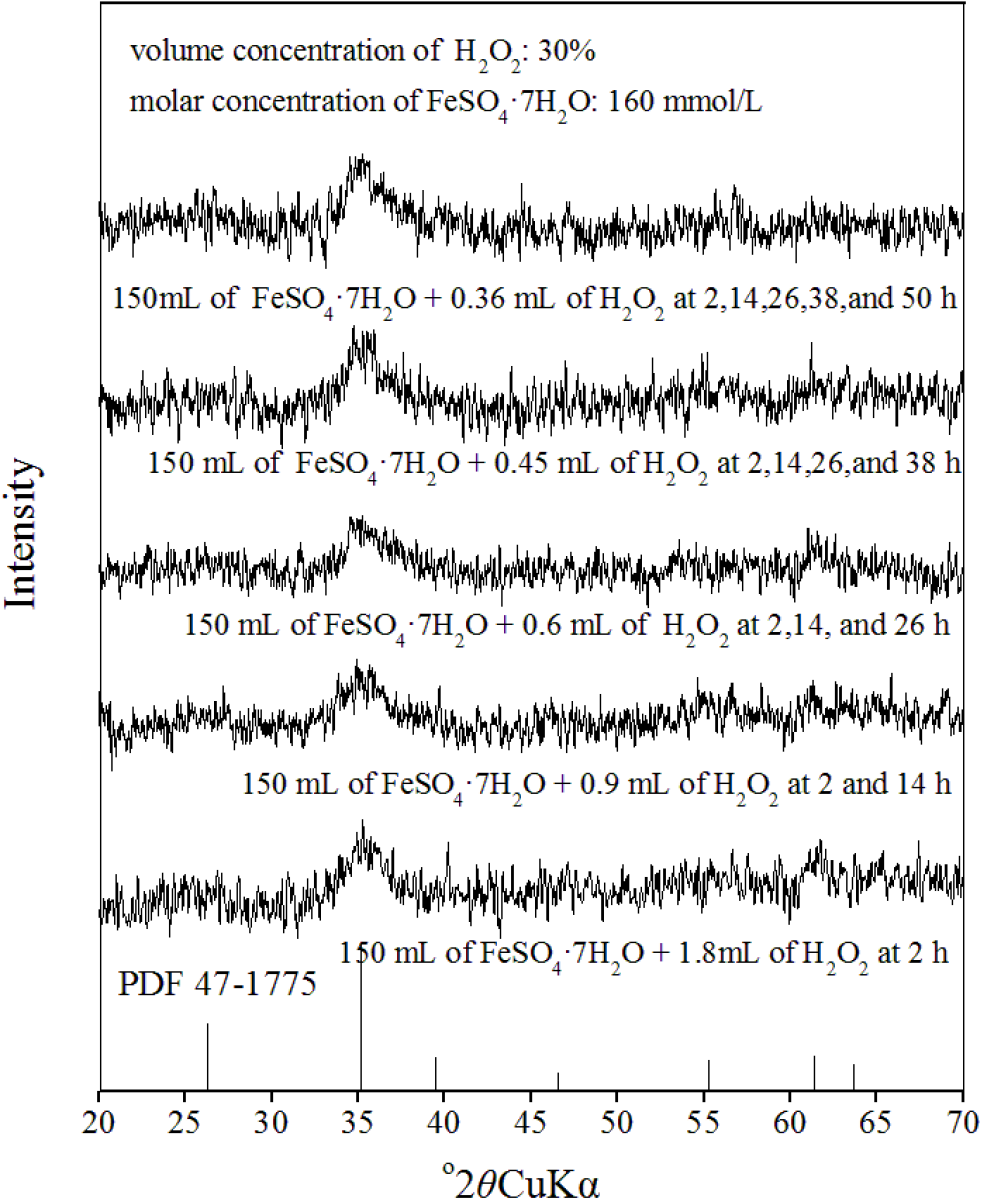
X-ray diffraction (XRD) patterns of iron precipitates harvested from ferrous ions oxidation systems with H_2_O_2_ adding FeSO_4_·7H_2_O solution at different rates

The mineral phase is primarily determined through XRD [28, 37]. The XRD patterns of the solid precipitates harvested from different treatments in this study are illustrated in Fig 4.

According to PDF 47–1775 [42] and the results of previous studies [26, 27], schwertmannite is the only mineral detectable in all synthesized products because the characteristic reflection peaks (2*θ* = 26.26°, 35.16°, 55.30°, 61.34°) are observed in their XRD patterns. The chemical formula of Schwertmannite can be described by Fe_8_O_8_(OH)_8-2x_(SO_4_)_x,_ where x ranges between 1 and 1.75 [33]. The Fe and S molar ratios of schwertmannite were 4.67, 4.75, 4.79, 4.98 and 5.04 when it was synthesized in treatment 1, treatment 2, treatment 3, treatment 4, and treatment 5, respectively. Thus, the chemical formula of schwertmannite harvested in above different systems can be expressed as Fe_8_O_8_(OH)_4.58_(SO_4_)_1.71_, Fe_8_O_8_(OH)_4.64_(SO_4_)_1.68_, Fe_8_O_8_(OH)_4.66_(SO_4_)_1.67_, Fe_8_O_8_(OH)_4.78_(SO_4_)_1.61_, and Fe_8_O_8_(OH)_4.82_(SO_4_)_1.59._ It is obvious that, the Fe and S molar ratio of schwertmannite slightly increased with slowing down the rate of H_2_O_2_ supply in the ferrous ions system.

**Fig 5:**
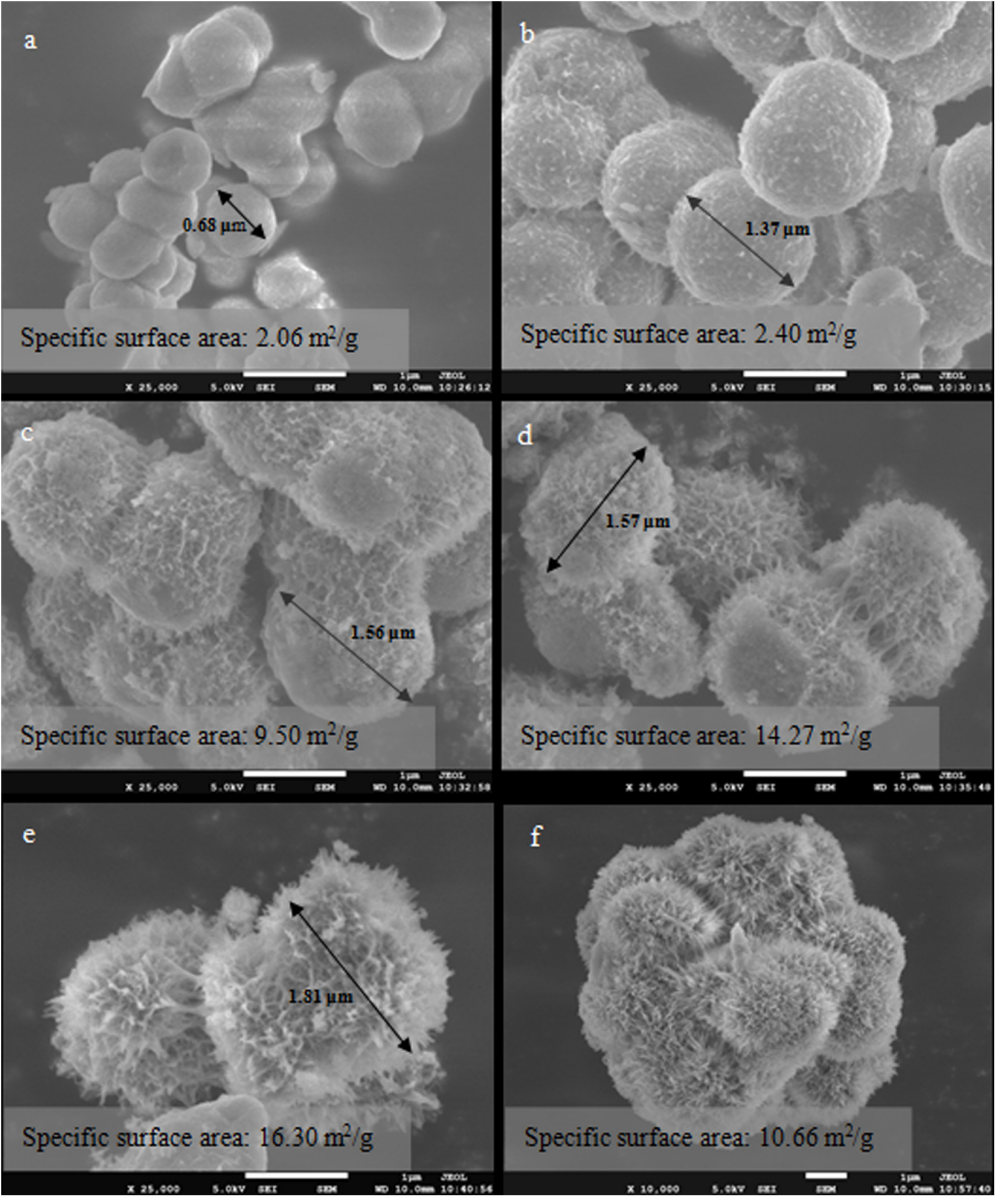
Scanning electron microscopy images and specific surface area of precipitates harvested from different schwertmannite synthesis systems. 150 mL of FeSO_4_·7H_2_O solution with addition of a: 1.80 mL of H_2_O_2_ at 2 h (treatment 1); b: 0.90 mL of H_2_O_2_ at 2 and 14 h (treatment 2); c: 0.60 mL of H_2_O_2_ at 2, 14, and 26 h (treatment 3); d: 0.45 mL of H_2_O_2_ at 2, 14, 26, and 38 h (treatment 4); e: 0.36 mL of H_2_O_2_ at 2, 14, 26, 38, and 50 h (treatment 5); and f: *Acidithiobacillus ferrooxidans*; volume concentration of H_2_O_2_: 30%; molar concentration of FeSO_4_·7H_2_O: 160 mmol/L

The morphology of schwertmannite was investigated through scanning electron microscopy (SEM) method, and the SEM images of the synthesized schwertmannite in this work are shown in Fig 5. Schwertmannite particles were small spheroids with a diameter of 0.68 μm (Fig 5A) and a specific surface area of 2.06 m^2^/g when formed in the fastest ferrous ions oxidation system, i.e., treatment 1. The size and specific surface area of these spherical particles were relatively smaller than that of schwertmannite formed under systems with slow H_2_O_2_ addition. For example, in treatment 2, the diameter and specific surface area of schwertmannite particles increased to 1.37 μm and 2.40 m^2^/g, and the particles surface evidently generated some spikes (Fig 5B). When the H_2_O_2_ supply rate was further reduced, the diameter or specific surface area of the schwertmannite particles further increased to 1.56~1.81 μm or 9.50~16.30 m^2^/g, and long needle-like structures grown on the particle surface formed the characteristic “hedge-hog” structure of schwertmannite (Fig 5C–5E). In general, small solid particle diameters indicate large specific surface area [43]. On the contrary, the results of the present study showed that the specific surface area of schwertmannite was increased with the increasing particle diameter. Therefore, the “spike” “needles”, and “hedge-hog” structures resulted in existence of more cavities on the schwertmannite surface, which may play a vital role in improving the specific surface area of schwertmannite. This finding had not been reported in previous studies. Consistent with the present study, schwertmannite “spherical” or “hedge-hog” structure had also been revealed in a number of previous studies [27, 28].

In addition, the specific surface area of schwertmannite in the present study was similar to that reported previously by Regenspurg et al. [27], Paikaray et al. [21], and Li et al.[29]. They found that the specific surface area of crystallized schwertmannite varied from 3.2~14.0 m^2^/g when schwertmannite is synthesized through the H_2_O_2_ oxidation of FeSO_4_ solutions. However, the factor that controls the morphology and specific surface area of schwertmannite during its chemical synthesis has not been explored in previous studies. In this study, the supply rate of the oxidant H_2_O_2_ was found to control the ferrous ions oxidation efficiency, and the total iron precipitation efficiency was determined to be a key factor regulating the particle size, morphology, and specific surface area of schwertmannite. In other words, slow H_2_O_2_ supply indicates low ferrous ions oxidation efficiency, low total iron precipitation efficiency, and large specific surface area of schwertmannite with many “hedge-hog” structures on its surface. To further confirm this finding, schwertmannite bio-synthesis by *Acidithiobacillus ferrooxidans* with a low ferrous ions oxidation efficiency in 160 mmol/L FeSO_4_·7H_2_O solution was conducted. It is found that, the bio-synthesized schwertmannite comprised “hedge-hog”-like particles (Fig 5F) with a specific surface of 10.66 m^2^/g. In addition, the schwertmannite particles aggregated more closely in the bio-synthesis systems than in the chemical synthesis systems, which may reduce the schwertmannite specific surface area to a certain extent. This phenomenon will be investigated in further studies.

### Arsenic(III) removal efficiency by schwertmannite harvested from schwertmannite chemical synthesis systems

**Fig 6:**
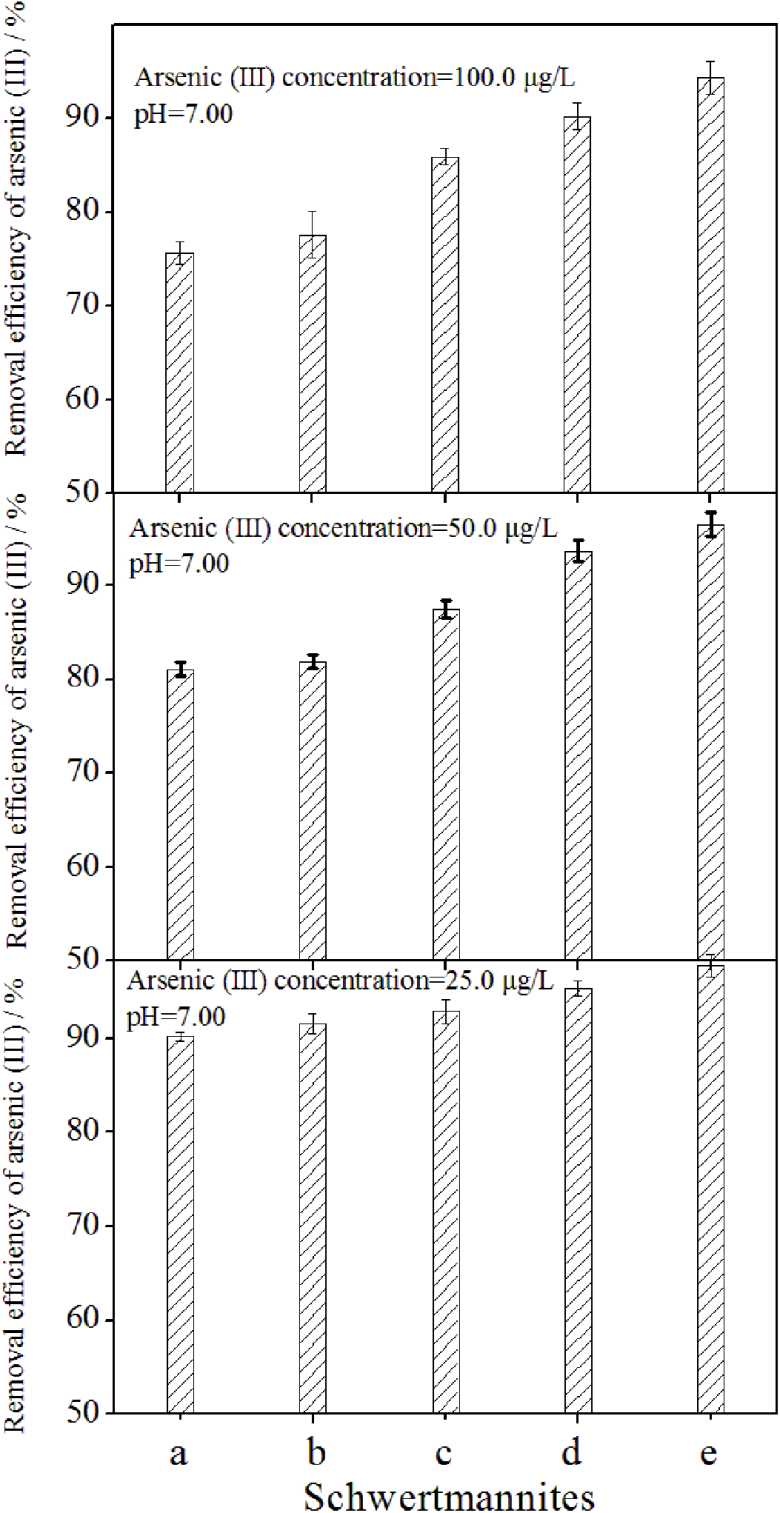
Removal efficiency for different concentrations of arsenic (III) in the solution over 0.25 g/L schwertmannite harvested from different chemical synthesis systems. 150 mL of FeSO_4_·7H_2_O solution with addition of a: 1.80 mL of H_2_O_2_ at 2 h (treatment 1); b: 0.90 mL of H_2_O_2_ at 2 and 14 h (treatment 2); c: 0.60 mL of H_2_O_2_ at 2, 14, and 26 h (treatment 3); d: 0.45 mL of H_2_O_2_ at 2, 14, 26, and 38 h (treatment 4); and e: 0.36 mL of H_2_O_2_ at 2, 14, 26, 38, and 50 h (treatment 5); volume concentration of H_2_O_2_: 30%; molar concentration of FeSO_4_·7H_2_O: 160 mmol/L.

The removal efficiency for different concentrations of Arsenic (III) in the neutral pH (~7.0) solution over 0.25 g/L schwertmannite harvested from different chemical synthesis systems is presented in Fig 6.

The arsenic (III) removal efficiency reached 90.2–97.8%, 81.1–96.5%, and 75.7–94.3% by schwertmannite with 2.06–16.30 m^2^/g specific surface area and 25, 50, and 100 μg/L of initial arsenic (III) contents, respectively, in the liquid system at pH ~7.0. In other words, when the specific surface area of schwertmannite increased by 6.91 times in this study, the arsenic (III) removal efficiency of schwertmannite increased 8.4%, 19.0%, and 24.8%, respectively, when the initial arsenic (III) concentrations were 25, 50, and 100 μg/L in contaminated water. It is noted that arsenic (III) removal efficiency exhibits a great difference with the variation of schwertmannite specific surface area at high arsenic (III) concentration level. In conclusion, schwertmannite with a small specific surface area had poor removal efficiency or low schwertmannite adsorption capacity for arsenic (III) in this research. These results are supported by the findings of Song et al. [23] and Paikaray et al. [21], who found that the maximum As (III) adsorption capacity of schwertmannite with a specific surface area of 325.5 m^2^/g is 45.50 mg/g at pH 3.0 [23], whereas that of schwertmannite with a specific surface area of 210.0 m^2^/g is only 20.1 mg/g [21] under the same condition. In addition, the price of FeSO_4_·7H_2_O powder and 30% (V/V) H_2_O_2_ are 200 RMB and 1, 500 RMB per ton. Combine the total iron precipitation efficiency (17.3%-29.3%) and chemical formula of schwertmannite obtained in different treatments in this study, the raw materials cost of schwertmannite synthesized through FeSO_4_ oxidation by H_2_O_2_ can be calculated as 5, 563–9, 508 RMB per ton. It is concluded that the raw materials cost of schwertmannite was great affected by total iron precipitation efficiency.

This technology described in this study can be used in the real practice for schwertmannite synthesis and arsenic removal whenever necessary due to the operation process is relatively simple. However, from the results of this study, it is concluded that the fast schwertmannite synthesis rate means small specific surface area and low schwertmannite adsorption capacity for arsenic (III). Therefore, the reasonable schwertmannite synthesis rate in practice need to be determined by the actual arsenic (III) content in contaminated-water. In other words, the schwertmannite synthesis efficiency and arsenic removal efficiency need to be overall consideration. This is a important issue need to be given more attention during this technology real practical implementation.

## Conclusions

Removal of arsenic (III) from groundwater by schwertmannite has attracted considerable attention in recent years because of its strong binding affinity to arsenite. To the best of our knowledge, this study is the first to address the effects of H_2_O_2_ supply rate, or ferrous ions oxidation efficiency, on the morphology and specific surface area of synthesized schwertmannite. Slow H_2_O_2_ supply rate indicated low ferrous ions oxidation efficiency thus low total iron precipitation efficiency in the schwertmannite synthesis systems. This characteristic increased the specific surface area of the synthesized schwertmannite by changing its morphology from spherical to “hedge-hog” structure. Moreover, schwertmannite with a large specific surface area exhibited a high removal efficiency for arsenic (III) from groundwater under neutral pH. The outcome of this study is valuable for the engineering application of synthesis of schwertmannite and its use in groundwater arsenic (III) removal.
